# An evolutionary model to predict the frequency of antibiotic resistance under seasonal antibiotic use, and an application to *Streptococcus pneumoniae*

**DOI:** 10.1098/rspb.2017.0679

**Published:** 2017-05-31

**Authors:** François Blanquart, Sonja Lehtinen, Christophe Fraser

**Affiliations:** 1 Department of Infectious Disease Epidemiology, Imperial College London, London, UK; 2 Big Data Institute, Li Ka Shing Centre for Health Information and Discovery, Nuffield Department of Medicine, University of Oxford, Oxford, UK

**Keywords:** antimicrobial resistance, fluctuating selection, microbiology, drug resistance, adaptation, balancing selection

## Abstract

The frequency of resistance to antibiotics in *Streptococcus pneumoniae* has been stable over recent decades. For example, penicillin non-susceptibility in Europe has fluctuated between 12% and 16% without any major time trend. In spite of long-term stability, resistance fluctuates over short time scales, presumably in part due to seasonal fluctuations in antibiotic prescriptions. Here, we develop a model that describes the evolution of antibiotic resistance under selection by multiple antibiotics prescribed at seasonally changing rates. This model was inspired by, and fitted to, published data on monthly antibiotics prescriptions and frequency of resistance in two communities in Israel over 5 years. Seasonal fluctuations in antibiotic usage translate into small fluctuations of the frequency of resistance around the average value. We describe these dynamics using a perturbation approach that encapsulates all ecological and evolutionary forces into a generic model, whose parameters quantify a force stabilizing the frequency of resistance around the equilibrium and the sensitivity of the population to antibiotic selection. Fitting the model to the data revealed a strong stabilizing force, typically two to five times stronger than direct selection due to antibiotics. The strong stabilizing force explains that resistance fluctuates *in phase* with usage, as antibiotic selection alone would result in resistance fluctuating behind usage with a lag of three months when antibiotic use is seasonal. While most antibiotics selected for increased resistance, intriguingly, cephalosporins selected for decreased resistance to penicillins and macrolides, an effect consistent in the two communities. One extra monthly prescription of cephalosporins per 1000 children decreased the frequency of penicillin-resistant strains by 1.7%. This model emerges under minimal assumptions, quantifies the forces acting on resistance and explains up to 43% of the temporal variation in resistance.

## 1. Introduction

The evolution of antimicrobial resistance in many pathogens is an important public health concern [1]. Resistance can evolve over time scales of a few months: fluctuations in the frequency of drug resistance have been observed in many important pathogens such as *Plasmodium falciparum*, a protozoan parasite causing malaria [2], and the bacterial species *Neisseria gonorrhoeae* [3], *Campylobacter* spp. [4], *Pseudomonas aeruginosa* [5], *Escherichia coli* [6], *Staphylococcus aureus* [6] and *Streptococcus pneumoniae* [7,8]. These fluctuations in levels of resistance may be due to rapid adaptation to a changing environment. The higher levels of resistance in the months when drug consumption is higher (e.g. wet season for antimalarial drugs in tropical regions, or winter for antibiotics in temperate regions) suggest direct selection for drug resistance due to drug consumption [2–6,8]. The reduction in levels of resistance when consumption is less intense suggests that resistance is counter-selected because it is costly. Few studies have explicitly linked patterns of fluctuating selection with data on antibiotic consumption. The few studies that have done so have performed direct correlation of frequency of resistance to consumption [8], or used more sophisticated ‘autoregressive integrated moving average’ (ARIMA) models [5,6]. These studies statistically support the link between resistance and consumption but do not lend themselves easily to a mechanistic or evolutionary interpretation. For instance, in all these studies, the fluctuations in resistance are in phase with fluctuations in consumption, or lag behind it by one to three months, but the interpretation for this lag is unclear. Furthermore, most of the time resistance is maintained at a relatively stable level in the long term, in spite of small seasonal fluctuations; this is unexpected under simple models that predict that the resistant or the sensitive strain will take over the population [9–11]. Last, resistance to a given antibiotic may evolve under the pressure of the multiple antibiotics that are consumed in the population, something which in general is not modelled. The first aim of the paper is to develop a generic model of adaptation to a fluctuating environment, applicable to a variety of settings and pathogens, in order to analyse longitudinal data on the frequency of resistance and antimicrobial consumption, and give a clear evolutionary interpretation to these patterns. We introduce these methodological developments, and we illustrate them in the context of antibiotic resistance in *S. pneumoniae*. The second aim is to learn about the population genetics of drug resistance in *S. pneumoniae* by applying the newly developed method.

*Streptococcus pneumoniae* is a commensal to humans carried mainly by children and the elderly (carriage is 30–40% in children [12]). It is asymptomatic most of the time, but may cause invasive diseases responsible for infections such as meningitis and pneumonia, causing approximately 800 000 death per year in children [13]. Multiple *S. pneumoniae* genotypes exhibiting resistance to antibiotics have emerged worldwide in past years [14,15]. Resistance, associated with globally distributed clones [16–19], has remained relatively stable in the USA and in Europe over the last 15–20 years [11,15,20]. At shorter temporal scales, the frequency of resistance exhibits small fluctuations, perhaps driven in part by seasonal fluctuations in antibiotic prescriptions [8,21]. The equilibrium level of resistance is variable across countries and correlates with levels of antibiotic use [21], suggesting antibiotics exert strong selection favouring resistant strains.

Here, we develop a generic model describing how fluctuations in antibiotic consumption, in particular seasonal fluctuations, drive fluctuations in antibiotic resistance [8]. Specifically, we do not describe either long-term trends within populations or large differences between populations, but rather focus on short-term fluctuations that occur between different months in a well-defined population. The model is rooted in the observation that in spite of fluctuating prescriptions, levels of resistance in *S. pneumoniae* only experience small fluctuations around a stable yearly average. We solve this model using a linearization method, leading us to naturally introduce an evolutionary force that stabilizes the frequency of resistance to an intermediate level. The model does not attempt to predict this intermediate equilibrium, which depends on the overall level of drug consumption in the population [21]. Under this minimal evolutionary model, fluctuations in resistance lag behind those in prescriptions. The new framework allows (i) quantification of the force that stabilizes resistance to intermediate levels, (ii) quantification of the sensitivity to antibiotic use, and (iii) dynamic modelling of the frequency of resistance over time. This framework is applicable to study temporal fluctuations in a variety of pathogens and resistances.

## 2. Methods

### (a) A general model for the evolution of resistance

Let us call *p_t_* the frequency of resistance to an antibiotic at time *t*, and *f* the function that determines the rate of change of the frequency of resistance as a function of the current frequency of resistance and the rates of antibiotic use. The dynamics of the frequency of resistance is given by a differential equation,2.1
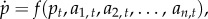
where the coefficients *a_i_*_,*t*_ are the rates of the use of antibiotic *i* and *n* is the number of antibiotics. The resistance under consideration can be any type of resistance (e.g. resistance to a specific antibiotic or multi-drug resistance). Resistance to one antibiotic may be statistically associated with resistance to other antibiotics. However, in the electronic supplementary material, we show that the dynamics of one type of resistance can be modelled independently of others (as assumed in equation (2.1)) if the associations between resistances are approximately constant.

We assume the frequency of resistance stabilizes to 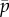 when all antibiotics are used at constant rates 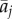, 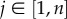. 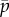 is considered a parameter of the model, and we do not attempt to predict the value of 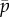 as a function of the average antibiotic consumption 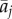. We choose the constant rates 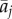 to be the temporal averages of use of each of these antibiotics. We consider temporal variations in antibiotic use as small perturbations around this average, leading to small perturbations in the frequency of resistance around its equilibrium frequency. The linearized system is given by the first-order Taylor series approximation of equation (2.1),2.2
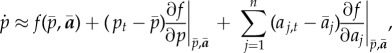
where 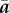 is the vector of the rates of antibiotic use. Because 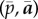 is an equilibrium, 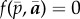; because this equilibrium is stable, 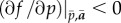. For notational simplicity, we rewrite equation (2.2) as2.3
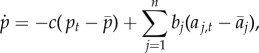
where 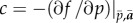 is positive, and 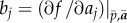. The parameters *b_j_* quantify the change in resistance frequency per unit time and per additional drug consumption unit, for each drug *j*. They represent the sensitivity of the change in resistance frequency to antibiotic use. The terms 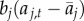 represent the response to antibiotic selection. The model accounts for association between different resistances. For example, penicillin resistance will increase under increased macrolide use if macrolide resistance is associated with penicillin resistance. The parameter *c* quantifies the strength of processes that stabilize the frequency of resistance to an intermediate value. In the following, we call the parameters *b_j_* ‘sensitivity to antibiotic use’, and the parameter *c* ‘stabilizing force’. Equation (2.3) emerges from linearization of any model that can be described with equation (2.1), and therefore can be re-derived for more specific epidemiological models (electronic supplementary material).

Equation (2.3) is a linear differential equation with temporally fluctuating coefficients that can be solved as2.4
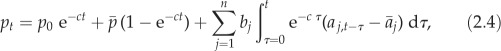
where *p*_0_ is the initial frequency of resistance. The first two terms in equation (2.4) represent transient effects that bring *p* to 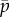. If the sensitivities of the change in frequency to antibiotic use are low (all *b_i_* are near 0), equation (2.4) simply expresses exponential decay of 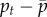 at a rate equal to *c*. The last term in equation (2.4) represents the impact of deviations from the average rate of antibiotic use on the frequency of resistance. The present frequency of resistance integrates all past fluctuations in selection due to variation in antibiotic use. Antibiotic use *τ* time units in the past imprints the present frequency of resistance with a weight e^−*cτ*^, such that more recent antibiotic use has more impact on the frequency of resistance. The imprint of past selective pressures depends on the stabilizing force. This is analogous to previous results on adaptation to a changing environment in a quantitative genetics modelling framework [22]. In the limit where the response to antibiotic selection (the terms 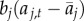) and the stabilizing force are both strong and of the same order, only the present antibiotic use affects the frequency of resistance, and equation (2.4) reduces to2.5
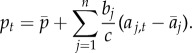


In this limit, thus, the excess frequency of resistance relative to the equilibrium value, 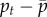, is a weighted sum of the excess antibiotic uses relative to the temporal average, 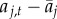. The weights are the ratios of the sensitivities over the stabilizing force, and the two forces cannot be identified separately. The weight *b_j_*/*c* is the change in equilibrium resistance frequency caused by a 1 unit increase in consumption of antibiotic *j*.

To get further analytical insights into equation (2.4), we describe the temporal change in antibiotic consumption as a sum of sinusoids with different periods, using Fourier transformation,2.6
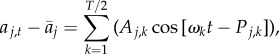
where the angular frequencies of the sinusoids are *ω_k_* = 2*πk*/*T*, where *T* is the number of data points, here *T* = 60 (12 points per year during 5 years). This transformation expresses the changes in antibiotic consumption around the average in a sum of sine waves, with successive periods *T*, *T*/2, *T*/3, … and 2 (in months), where the amplitudes *A_j_*_,*k*_ and phases *P_j_*_,*k*_ are functions of the *a_j_*_,*t*_ (electronic supplementary material).

The contribution of each sinusoid depends on the amplitude *A_i_*_,*j*_. Here, the sinusoid with largest amplitude will often be that with period 12 months as antibiotic use is seasonal (use is more frequent in winter than in summer).

Plugging equation (2.6) into equation (2.4), solving the integral and neglecting transient initial effects, we obtain2.7
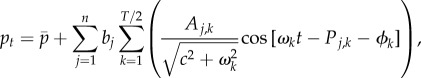
with *ϕ_k_* = atan[*ω_k_*/*c*].

The fluctuations in the frequency of resistance mirror those in antibiotic use. To each sinusoid of the fluctuations in antibiotic use, with amplitude *A_j_*_,*k*_ and phase difference *P_j_*_,*k*_, is associated a sinusoid of the fluctuations in frequency of resistance, with amplitude 
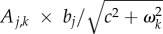
 and phase difference *P_j_*_,*k*_ + *ϕ_k_*. The amplitude of fluctuations in the frequency of resistance increases with the sensitivity *b_j_* and decreases with *c*. These effects are easily understood, as antibiotic use tends to push the frequency of resistance away from the equilibrium value 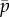, while the stabilizing force brings it back to 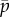.

**Figure 1.**
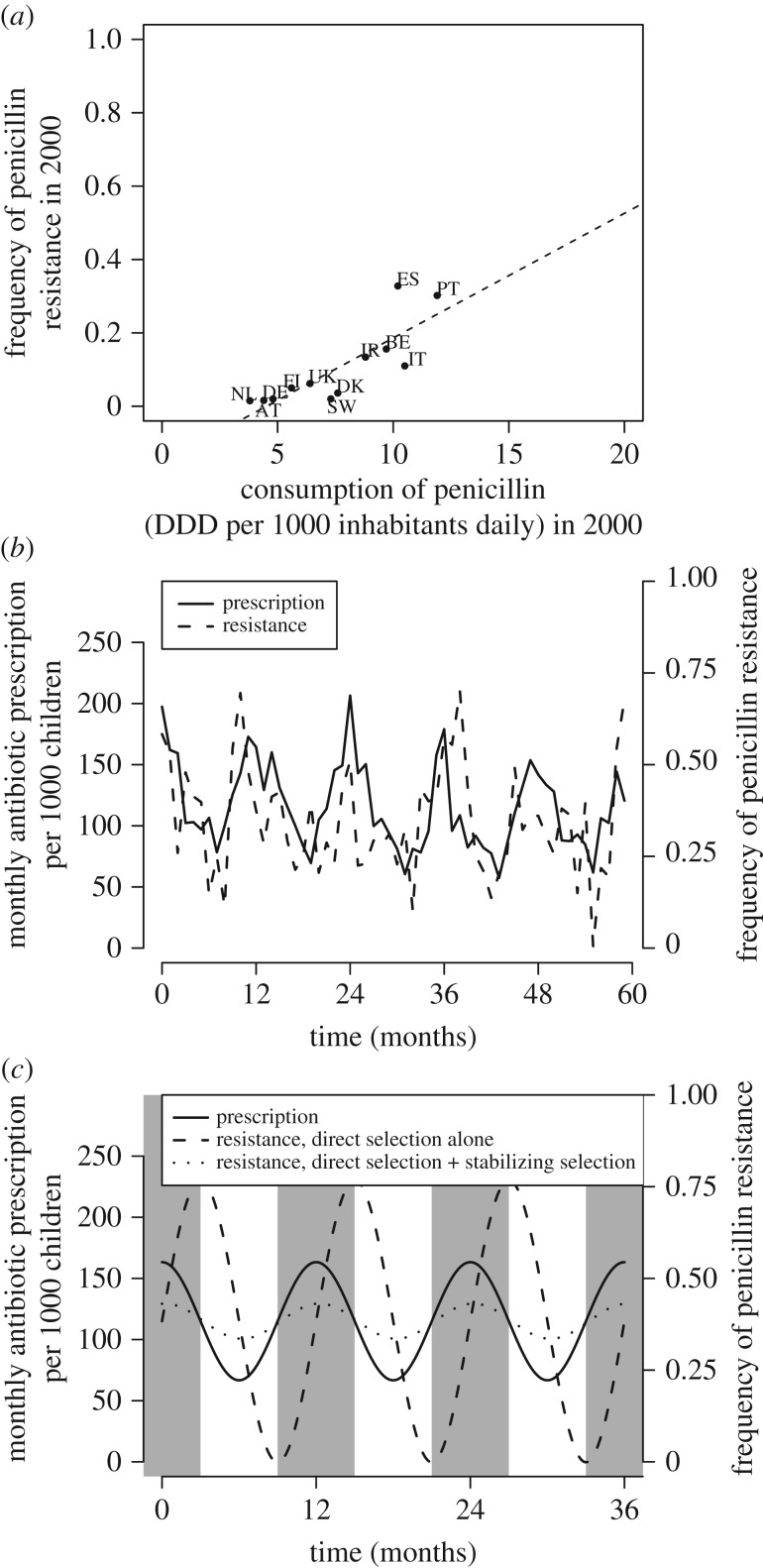
(*a*) The average frequency of penicillin non-susceptibility in several European countries in 2000 correlates with the average consumption of penicillin in defined daily dose (DDD) in these countries in 2000 (*R*^2^ = 0.67) (data available on the ECDC website). (*b*) The seasonal dynamics of penicillin consumption and penicillin non-susceptibility in a Jewish community in Israel [8]. (*c*) An evolutionary model of seasonal fluctuations in antibiotic prescription (plain line) and resistance frequency (dashed and dotted lines). Grey and white zones highlight the winter and summer months. Resistance frequencies were obtained from equation (2.7), with *b* = 0.05 and *c* = 0 (dashed line), and *b* = 0.05 and *c* = 50 (dotted line). A stronger stabilizing force reduces the amplitude and the phase difference of fluctuations in resistance frequency.

The phase difference *ϕ_k_* is positive, indicating that fluctuations in the frequency of resistance lag behind seasonal fluctuations in antibiotic use. The lag is larger for the more rapid sinusoids (those with a small *ω_k_*), as it is harder for the population to keep track of rapidly changing environments. Interestingly, the lag is smaller as the stabilizing force gets stronger. In the limit where there is no stabilizing force, when antibiotic use is simply given by a sine wave with period 12 months, the phase difference is *π*/2 (i.e. resistance frequency lags three months behind antibiotic use; figure 1). This result is not specific to our framework: rather, it is a fundamental consequence of antibiotic treatment affecting the *change* in frequency 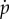 and not the frequency itself. In other words, when antibiotic use is maximal (e.g. in December), the *increase* in frequency is maximal. The frequency of resistance peaks when the treatment rate passes the threshold under which resistance is no longer selected for. In the limit of 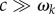 for all *k*, phase difference tends to 0 and we recover the result of equation (2.5),2.8
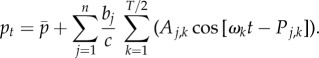

These results show that the amplitude of temporal fluctuations in resistance, and the phase difference with fluctuations in antibiotic use, allow in principle inference of both the sensitivity to antibiotic use and the stabilizing force.

### (b) Application to data on the evolution of resistance in *S. pneumoniae*

We applied this model to published data on monthly antibiotic prescription and monthly frequency of resistance in *S. pneumoniae* from 1999 to 2004, before the pneumococcal vaccine was introduced in the region. The data were collected in children less than 5 years old of Jewish and Bedouin communities living in Israel [8]. Prescription data, for a total of 236 466 prescriptions, were collected in seven paediatric primary care clinics, five in Jewish urban centres and two in Bedouin townships, representing prescription to approximately 20% of the region's children below 5 years of age. Prescriptions of four antibiotics were recovered: amoxicillin and amoxicillin–clavulanate (penicillin family), oral cephalosporins (β-lactam family, including cefaclor, cephalexin monohydrate and cefuroxime-axetil) and azithromycin (macrolide family). The use of other antibiotics was negligible [8]. Bacterial isolates were sampled for children presenting with acute otitis media, which is commonly caused by *S. pneumoniae*. Among 11 022 samples, *S. pneumoniae* was recovered in 1401 Jewish children and 2205 Bedouin children with acute otitis media. Pneumococcal isolates were then tested for resistance to sulfamethoxazole, tetracycline, erythromycin, clindamycin and chloramphenicol using the Kirby–Bauer disc diffusion method as described in [23]. Resistance to penicillin was determined using minimum inhibitory concentration (MIC) assays, where a strain is considered resistant when MIC > 1.0 µg l^−1^. Three types of resistances are considered here: penicillin resistance, erythromycin resistance (macrolide) and multi-drug resistance, defined as resistance to three antibiotic classes or more (often including penicillin and erythromycin resistance). Data are available in [8].

We used these data to model the dynamics of each type of resistance as a function of the use of the antibiotics. Note that the different models for the different types of resistance are not necessarily independent, because different resistances may be associated. We assumed antibiotic use was proportional to prescription, such that we could directly use antibiotics prescriptions to reflect the coefficients *a_i_*_,*t*_. For a set of parameters 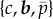 (where ***b*** is the vector of sensitivities to different antibiotics), we computed the predicted frequency of resistance over time 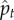 using equation (2.7). We fitted the model separately in the Bedouin and Jewish children. The likelihood was given by the product over all months of the probability that *p_t_N* samples were resistant if the true frequency of resistance was 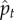:2.9
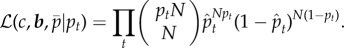


In the absence of other information, the number of samples was assumed to be the same each month, and equal to *N* = 23 for Jewish children and *N* = 37 for Bedouin children (1401 and 2205 samples over 60 months). We found maximum-likelihood estimates of the parameters using the Nelder–Mead optimization method. Confidence intervals were derived using Markov chain Monte Carlo sampling of the likelihood function.

**Figure 2.**
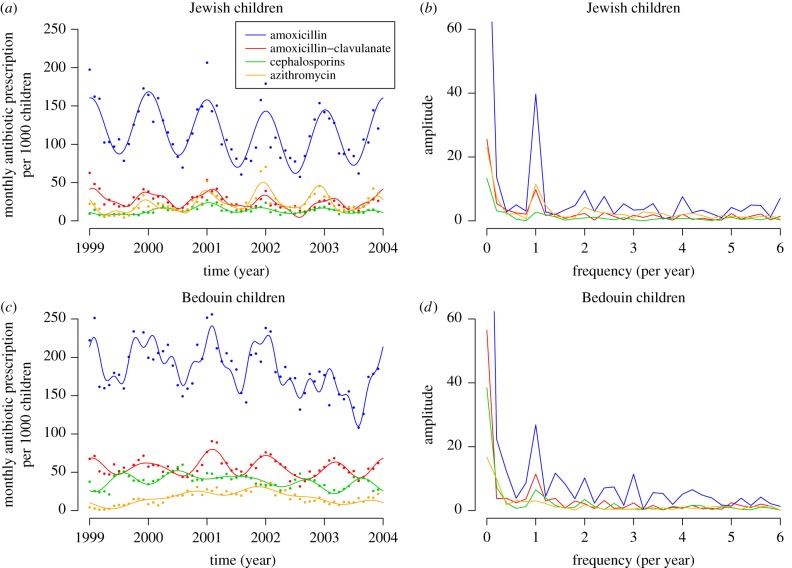
Monthly antibiotic prescriptions in (*a*,*b*) Jewish and (*c*,*d*) Bedouin children. (*a*,*c*) The time series, with the raw data (dots) and the Fourier series approximation of the time series *a_i_*_,*t*_ (curves). (*b*,*d*) The frequency spectrum for each antibiotic, in the two communities. The peak in amplitude at frequency 1 per year demonstrates the marked seasonality of prescriptions.

We Fourier-transformed the time series of antibiotic prescription, and only used the sinusoids of greatest amplitude (those explaining the largest fluctuations in the data). Specifically, we retained the minimum number of sinusoids such that the coefficient of determination *R*^2^ between the data and the Fourier series was greater than 0.7. This ensures that prescription data were correctly described but that smaller fluctuations due to sampling error were not fitted (figure 2). Ideally, one would want to explicitly take into account uncertainty on monthly prescription rates in the model. However, we did not have access to data on the absolute counts of prescriptions per month, only to prescriptions per 1000 children. Last, we did not use data on amoxicillin–clavulanate in the analysis as it was strongly correlated with amoxicillin prescription (electronic supplementary material, figure S2).

## 3. Results

We consistently detected a strong stabilizing force on antibiotic resistance, both in the Jewish population and in the Bedouin population (electronic supplementary material, table S1: when inferring the parameters from equation (2.7)). The stabilizing force (the parameter *c*) was always much larger than the frequencies *ω_j_* = 2*πj*/*T* of the sinusoids capturing fluctuations in antibiotic use (which ranged from *π*/30, for the sinusoid of period 60 months, to *π*/2, for that of period 4 months). Thus, the sensitivities and the stabilizing force were not identifiable. As a result, likelihood mainly depended on the ratio *b_i_*/*c*, such that likelihood values similar to the maximum likelihood can be obtained for a wide range of *b_i_* and *c* values. We thus approximated the dynamics of resistance using equation (2.8).

**Figure 3.**
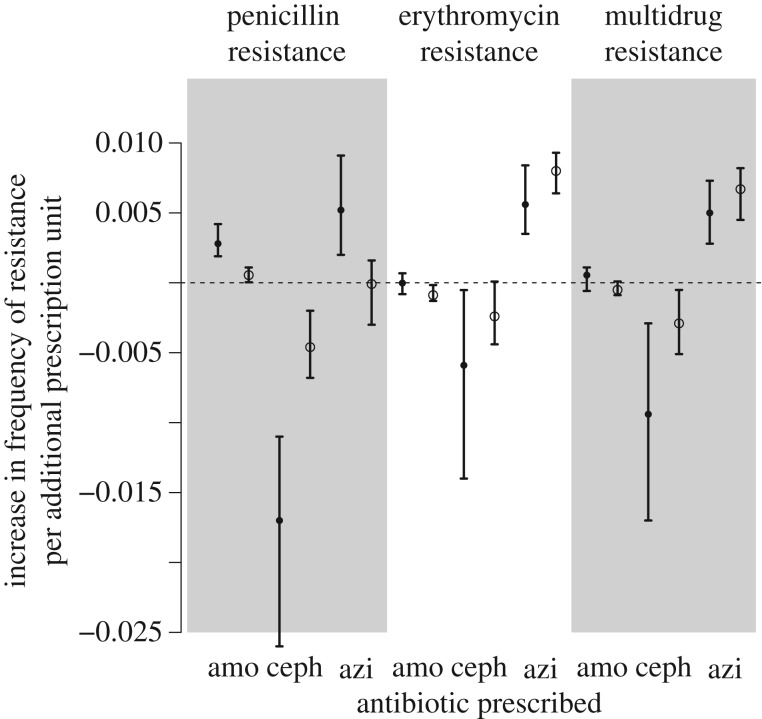
Effect sizes and 95% CIs for the ratios *b*_amo_/*c*, *b*_ceph_/*c* and *b*_azi_/*c*, for the population of Jewish children (plain dots) and Bedouin children (open points). The model was fitted independently on Jewish and Bedouin children.

Fitting equation (2.8) to the data revealed significant response to antibiotic use in both populations, as quantified by the maximum-likelihood estimates of the ratios *b_i_*/*c* (table 1 and figure 3). These ratios *b_i_*/*c* represent the increase or decrease in the equilibrium frequency of resistance caused by a 1 unit (1 prescription per 1000 children per month) increase in prescription of the corresponding antibiotic. As expected, amoxicillin use selected for penicillin resistance in both populations. Azithromycin selected for erythromycin resistance in both populations, and for multi-drug resistance in both populations. Curiously, equilibrium levels of resistance in Bedouin children were lower than in Jewish children, in spite of higher rates of prescription. Accordingly, the sensitivity to antibiotic use was generally smaller in Bedouin children (see Discussion).

**Table 1.** Maximum-likelihood parameter estimates and 95% confidence intervals for the reduced model. For each combination of type of resistance and population, we show maximum-likelihood estimates and 95% confidence intervals of the ratios *b*_amo_/*c*, *b*_ceph_/*c* and *b*_azi_/*c*. Coefficients in italics are significantly different from 0. We also show the maximum-likelihood estimate of the stable frequency of resistance 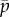, if all antibiotics were used at their average value, maximum likelihood and the coefficient of determination *R*^2^ of the model.

resistance	population	*b*_amo_/*c*	*b*_ceph_/*c*	*b*_azi_/*c*	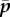	ML	*R*^2^
penicillin	Jewish	*0.0028* (0.0019; 0.0042)	−*0.017* (−0.026; −0.011)	*0.0052* (0.002; 0.0091)	0.35	−147.1	0.43
penicillin	Bedouin	*0.00055* (4 × 10^−5^; 0.0011)	−*0.0046* (−0.0068; −0.002)	−8.5 × 10^−5^ (−0.003; 0.0016)	0.23	−160.6	0.16
erythromycin	Jewish	−1.8 × 10^−5^ (−0.00081; 0.00068)	−*0.0059* (−0.014; −0.00052)	*0.0056* (0.0035; 0.0084)	0.24	−128.6	0.24
erythromycin	Bedouin	−*0.00088* (−0.0013; −0.00016)	−0.0024 (−0.0044; 8.7 × 10^−5^)	*0.008* (0.0064; 0.0093)	0.16	−163.7	0.31
multi-drug	Jewish	0.00055 (−0.00058; 0.0011)	−*0.0094* (−0.017; −0.0029)	*0.005* (0.0028; 0.0073)	0.19	−130.7	0.21
multi-drug	Bedouin	−5 × 10^−4^ (−0.00088; 9.4 × 10^−5^)	−*0.0029* (−0.0051; −0.00051)	*0.0067* (0.0045; 0.0082)	0.19	−151.2	0.25

Our analysis also revealed that a given antibiotic may select for multiple resistances, most of them consistent in the Jewish and Bedouin populations. Azithromycin selected for penicillin resistance in the Jewish population. This is in accordance with most azithromycin-resistant isolates also being penicillin resistant [24], such that selecting for azithromycin resistance indirectly selects for penicillin resistance. Similarly, azithromycin selected for multi-drug resistance in both populations, in accordance with most azithromycin-resistant isolates also being multi-drug resistant [24]. Intriguingly, cephalosporins strongly selected against penicillin, erythromycin and multi-drug resistance, in both populations. This suggests that the cephalosporin-resistant strains are penicillin-susceptible and erythromycin-susceptible (see Discussion).

From equation (2.3), the typical strength of the stabilizing force over antibiotic selection is the ratio 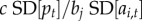, where 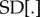 denotes the temporal standard deviation. For any resistance with a significant effect, this ratio ranged in absolute value from 1.7 to 5.

Our results were robust to the modelling choice of temporal fluctuations in prescription: similar results were obtained when we fitted cubic splines to the data, instead of Fourier series (electronic supplementary material, figure S3). Moreover, when the response to antibiotic selection and the stabilizing force are both strong and of the same order, the ratios *b_i_*/*c* can also be recovered using linear regression of the frequency of resistance onto the prescription rates of antibiotics (equation (2.5)). Using this alternative method, we also recovered similar coefficients (electronic supplementary material, figure S4).

**Figure 4.**
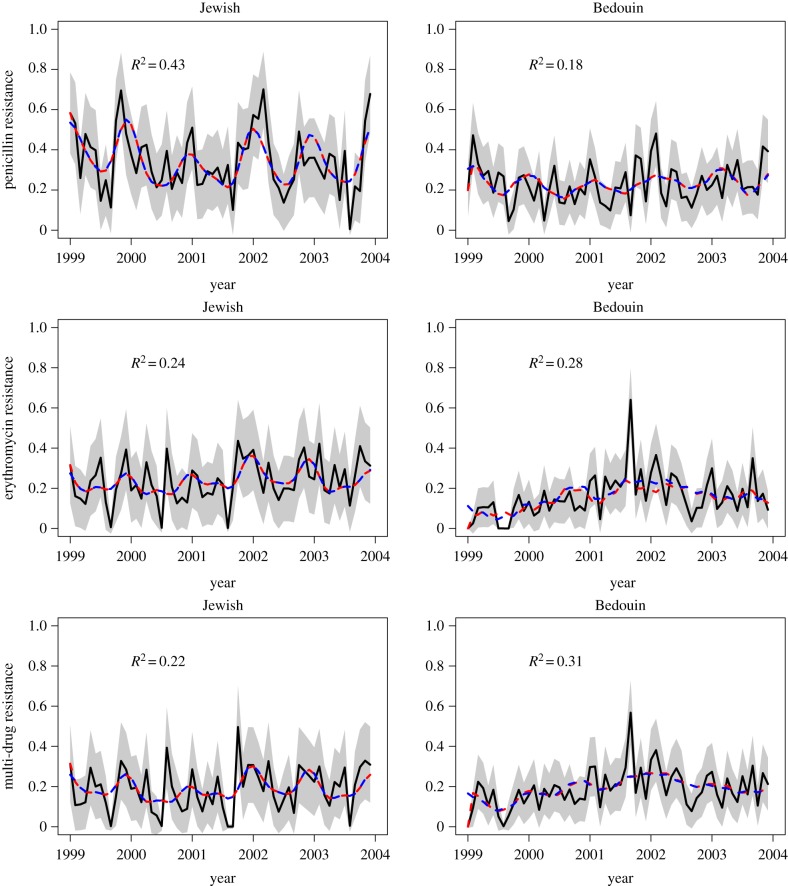
Predicted frequency of resistance as a function of time, for the Jewish (left) and Bedouin (right) populations. Black lines are the data with 95% confidence intervals as a grey region; dashed red lines are the predictions from the full model (equation (2.7)) with maximum-likelihood parameters (electronic supplementary material, table S1); dashed blue lines are the predictions from the approximation (equation (2.8)) with maximum-likelihood parameters (table 1) and can hardly be distinguished from the red line.

Finally, our fitness model predicted the fluctuations in antibiotic resistance well, with a coefficient of determination ranging from 16% to 43% (table 1 and figure 4). The dynamics of multi-drug resistance were highly correlated with those of erythromycin resistance. For penicillin resistance in the Jewish children, where the coefficient was highest at 43%, we assessed the predictive power of our model by fitting the model on the data from the first 4 years (‘training dataset’), then predicting the frequency of resistance in the fifth year. With this approach, we predicted 38% of the temporal variation in the frequency of resistance in the fifth year. Thus, our model is able in some cases to accurately predict the short-term evolution of resistance from prescription data.

## 4. Discussion

We developed a predictive model of the evolution of antibiotic resistance under changing antibiotic consumption. This model is applicable to any pathogen and any type of resistance, and requires finely resolved temporal data on antibiotic consumption and the frequency of resistance. Because antibiotic consumption selects for resistance, temporal fluctuations in resistance mirror temporal fluctuations in antibiotic consumption. The amplitude and phase difference of fluctuations in resistance depend on the direct selection due to antibiotics and the stabilizing force that acts on resistance, making it possible to quantify the strength of these two important drivers of resistance evolution. Specifically, resistance is generally expected to lag behind fluctuations in antibiotic consumption. Resistance directly correlates with antibiotic consumption only in the limit when the stabilizing force is strong. Indeed, under a simple model with no stabilizing force, the increase in resistance would be maximal during the month of highest use, introducing a lag between antibiotic consumption and frequency of resistance. For example, when antibiotics are seasonally prescribed with a higher rate in colder months, with no stabilizing force we expect the frequency of resistance to increase during winter months (e.g. October–March) and to decrease in summer months (e.g. April–September), such that resistance lags three months behind fluctuations in antibiotic use.

We applied the model to pneumococcal resistance in two communities followed monthly over 5 years in Israel. Sixteen to 43 per cent of the temporal variation in antibiotic resistance was attributable to fluctuations in antibiotic prescriptions; some of the rest may be stochastic noise, or attributable to other causes. Our results broadly confirm what we know about the biology of antibiotic resistance in *S. pneumoniae*, but now give quantitative estimates of the forces that govern pneumococcal evolution.

(i) A strong force, typically two to five times stronger than direct selection due to antibiotics, maintained an intermediate frequency of resistance in these communities. This force implies that resistance is always at equilibrium between the stabilizing force and antibiotic selection, which explains why there is no lag between consumption and resistance. This force was anticipated but never quantified. Indeed, levels of resistance in *S. pneumoniae* are stable across a wide range of antibiotic use, and robust to major ecological changes such as the introduction of the vaccine [25,26]. This suggests a strong evolutionary force maintain the stable frequency of resistance in this species. However, the nature of this force is unexplained. Resistant and sensitive strains are ecologically similar and compete for the same hosts, such that the fittest strain (the resistant or the sensitive depending on the balance between the rate of antibiotic treatment and the cost of resistance) is predicted to take over the population [10,11,27]. The problem of coexistence of multiple sensitive and resistant strains was also noted for other pathogens with similar life cycles, for example, *S. aureus* [9]. Recently, it has been proposed that resistant strains may be maintained by epistasis between loci conferring resistance and loci conferring longer carriage duration, generating linkage disequilibrium between these loci [28]. Accordingly, in *S. pneumoniae*, resistance is associated with capsular types with longer carriage [28], and may also be associated with other genetic factors causing longer carriage duration [29]. Other mechanisms such as host population structure may additionally contribute to coexistence.
(ii) Penicillin use selected for penicillin resistance, and azithromycin use selected for erythromycin resistance. Amoxicillin use selected for penicillin resistance only, and did not select for resistance to other drugs. Azithromycin selected for erythromycin resistance and favoured multi-drug resistance, probably because most erythromycin-resistant strains are multi-drug resistant [8]. Most of the coefficients describing sensitivities to antibiotic use were consistent in the two populations, in spite of distinct prescription patterns. Actually, we found similar coefficients when inferring the same set of coefficients jointly for the Jewish and Bedouin populations (electronic supplementary material, table S3, figure S5). The model with a single set of coefficients for both populations performed similarly in terms of Akaike information criterion (AIC) for erythromycin resistance (AIC was 600.6 for the separate model, and 599.6 for the joint model) and for multi-drug resistance (AIC was 579.8 for the separate model and 581.2 for the joint model). For penicillin resistance, the separate model was strongly supported compared with the joint model (AIC was 631.4 for the separate model, 656.4 for the joint model), probably because in the Bedouin population penicillin resistance did not evolve in response to azithromycin treatment (figure 3).

Illustration of the framework using a specific epidemiological model predicted that the sensitivity to antibiotic use is 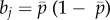 when considering a resistant strain versus a sensitive strain (electronic supplementary material). We expect this result to be quite general, as the term 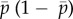 is the genetic variance, to which the response to selection is proportional. We did not have power to estimate *b_j_* separately from the stabilizing force *c* in most cases, because *c* was strong. However, encouragingly, when *c* was relatively small so that *b_j_* could be accurately measured, when considering the evolution of erythromycin resistance in Bedouin children, we had *b_azi_* = 0.11 and 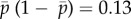 (electronic supplementary material, table S1).

Our model did not include the explicit pharmacodynamics of the drugs in the human body. Instead, treatment was assumed to instantaneously clear sensitive infection. If we were to relax this assumption, we would expect drugs with longer half-life (such as erythromycin [23]) to exert a stronger selective pressure for a given dose, which will translate into stronger sensitivities in our model. Accordingly, sensitivity to azithromycin selection was stronger than to penicillin selection (figure 3).

However, two results are biologically less intuitive. First, resistance was lower in Bedouin in spite of higher antibiotic consumption, and accordingly, the sensitivity was smaller for Bedouin children (table 1). This relative lack of response of the Bedouin population was noted before [8]. Four phenomena could explain this pattern. As the national healthcare plan started in 1996, the Bedouin population was exposed to antibiotics starting only a few years before the study, such that resistance may not be at its equilibrium yet. This is not a plausible explanation for lower resistance in the Bedouin children, as our model indicates resistance reacts fast to changes in antibiotics prescription, and the sensitivity to antibiotic use is also lower in Bedouin children than in Jewish children. The Jewish children may be prescribed antibiotics in other (private) clinics, while the Bedouin children are only prescribed antibiotics in the General Health Insurance Plan clinics where the data were collected. This is a possibility, as 90% of Bedouin but only 60% of Jewish children benefit from the national insurance [23]. Carriage of *S. pneumoniae* may be larger in Bedouin adults than in Jewish adults because of overcrowding [8]. The pneumococcal population in adults is under reduced antibiotic selection, which could overall limit the evolution of resistance in the Bedouins. Finally, the Bedouin children may have lower adherence to antibiotic treatment.

Second, the use of cephalosporins decreased the frequency of penicillin and erythromycin resistance, an effect present in the two communities. In the Jewish community, for example, one more prescription of cephalosporins per 1000 children resulted in a 1.7% decrease in the frequency of resistance to penicillin. Some of these effects were already detected in the original publication presenting the data, which used linear regressions of resistance over prescription to reveal antibiotic selection [8]. However, the negative impact of cephalosporins on resistance was previously only detected for resistance to penicillin in Bedouin children, and we suggest here that this effect is more general, as it was present in Jewish children as well, and both for penicillin and azithromycin resistance. The significance of this effect in both communities (where cephalosporin and amoxicillin use are not temporally correlated in the same way; electronic supplementary material, figure S1) suggests this effect is genuine, and not only an artefact caused by the inverse seasonality of cephalosporin use in Bedouin children (cephalosporins are prescribed more frequently in summer because they are used to treat skin and soft tissue infections that are more frequent in summer [8]). Moreover, the effect of cephalosporins in reducing resistance is not due to our specific framework as it was also present in a simple regression of resistance on antibiotic prescription (electronic supplementary material, figure S4). This result could be explained if cephalosporin-resistant strains tend to exhibit penicillin *intermediate* resistance (MIC 0.125–1 µg l^−1^), such that cephalosporin prescription reduces the frequency of penicillin *full* resistance (MIC > 1 µg l^−1^). Accordingly, in Israel, cephalosporin resistance was more frequent among penicillin-intermediate than among penicillin-susceptible (MIC < 0.125 µg l^−1^) pneumococcal isolates [30], and children with penicillin-intermediate pneumococcal acute otitis media showed a greater rate of failure of treatment by cephalosporins [24,30]. Further supporting this hypothesis, several strains highly resistant to cephalosporins but only intermediately resistant to penicillin have been isolated in the USA [31], South Africa [32] and Japan [33,34], suggesting a trade-off between cephalosporin and penicillin resistance. Cephalosporin resistance requires altered affinity to cephalosporins of the penicillin-binding proteins (PBP) 1a and 2x, while intermediate to high penicillin resistance requires reductions in the affinities of PBP 1a, 2x and 2b. Thus, strains with altered affinities in PBP 1a and 2x (but not 2b) will present high cephalosporin resistance but low to intermediate penicillin resistance [14,31,34].

### (a) Comparison with spatial data on antibiotic consumption and resistance

The ratios *b_i_*/*c* inferred in this study are the increase or decrease in the equilibrium frequency of resistance caused by a 1 unit (1 prescription per 1000 children) increase in prescription of the corresponding antibiotic. These ratios are directly comparable with the regression coefficients obtained by performing a multi-variate regression of frequency of a resistance over consumption of various antibiotics across countries in Europe [21] (figure 1). In the European data, prescription is given in defined daily dose (DDD) per 1000 inhabitants per day. Thus, one prescription unit in the Israel data analysed here is equivalent to 10/30 = 0.33 prescriptions in the European data, assuming a prescription is 10 DDD [35,36]. After this correction, the multi-variate regression of resistance over prescription for penicillin gives a coefficient of 0.004 (per prescription per 1000 children) on average across years, comparable with that found for Jewish children at 0.0028 per prescription per 1000 children (table 1).

### (b) Connection with previous evolutionary models

Our model bears connection with classical evolutionary models of adaptation to a changing environment. Classical models made it clear that fluctuating selection alone cannot maintain genetic polymorphism in the long run [37,38]. In a different framework, considering a continuous trait evolving under fluctuating stabilizing selection, Lande & Shannon [22] derived results analogous to our equation (2.4) (see their equation (2.7)). They show, similarly, that the current trait is the result of all past selective pressures, with more recent environments having more impact on the present trait, and that environments experienced more recently matter all the more relative to past ones with respect to strength of stabilizing selection. In our model, polymorphism is not maintained by a balance between mutation and stabilizing selection as in Lande & Shannon's quantitative genetics model, but rather by a stabilizing force that emerges naturally from our perturbation approach, and could be generated by a variety of distinct mechanisms, such as immigration from a larger population (electronic supplementary material) or epistasis with loci controlling the duration of carriage [28].

### (c) Simple models versus complex models

In the simple models outlined above, the evolution of the frequency of resistance depends on this frequency alone (equation (2.1)). However, in more complex models, the evolution of the frequency of resistance may depend on multiple variables. When the rate of antibiotic use is not the same in different classes of the population (e.g. age structure), the evolution of the overall frequency of resistance needs to be modelled by tracking the evolution of the frequencies in each class. Similarly, when the dynamics of resistance are associated with the evolutionary dynamics of linked loci, for example, loci determining the capsular type, the dynamics of the frequencies at different loci and the linkage disequilibrium between them need to be modelled. The same approach could in principle be extended to account for these more complex models, if more detailed data are available. For example, one can model fluctuations in the frequency of resistance in two classes of individuals around equilibrium with the same perturbation approach. But complex models would have more unknown parameters and the structure of such models would be uncertain, as we have limited knowledge of the processes that maintain coexistence in *S. pneumoniae* and other species. A simple phenomenological model may be used as an alternative, as it has a clear interpretation, can readily be fitted to data and is applicable to any pathogen species, allowing cross-species comparisons.

### (d) Public health implications of the strong stabilizing force

The existence of a strong stabilizing force on antibiotic resistance has public health implications. Because the frequency of resistance is always at equilibrium with the stabilizing force, there is no lag between antibiotic consumption and the frequency of resistance. Consequently, any change in antibiotic consumption will have an immediate effect on the frequency of resistance, which is positive from a public health perspective if antibiotic consumption is reduced. For example, in France, a nationwide campaign launched in 2002 resulted in a 26.5% reduction in overall community antibiotic use [39], immediately reducing the frequency of resistance in *S. pneumoniae* [40]. This contrasts with a situation where antibiotic consumption would have to be reduced below a threshold before resistance starts to decrease [41]. Making longer-term predictions would require inferring the levels of antibiotic consumption below which full antibiotic sensitivity evolves. Unfortunately, we cannot address this question within our framework, but more mechanistic modelling or cross-country comparisons might help answer it.

## 5. Conclusion

We developed a framework to dynamically model the evolution of resistance in the presence of temporally fluctuating rates of antibiotic use. This method has several advantages compared with what would perhaps be the most natural approach—correlating the frequency of resistance to antibiotic prescription rates. It quantifies the stabilizing force as well as the response to antibiotic selection, which is important because the stabilizing force determines the lag between changes in antibiotic use and the frequency of resistance. It is valid under minimal assumptions, yet has a clear link with dynamical models of evolution of resistance—in fact, more specific mechanistic models may be special cases of our generic framework. Application of the framework to data on two communities in Israel revealed a very strong stabilizing force in *S. pneumoniae* in Israel and, intriguingly, that increased cephalosporin prescription reduces the frequency of resistance to both penicillins and macrolides. The model explains 16–43% of the temporal variation in the frequency of resistance. The evolutionary-based modelling introduced here may be an interesting tool to predict the evolution of resistance in a variety of pathogens, and to guide public health policies.

## Supplementary Material

Supplementary Text, Figures and Tables

## Supplementary Material

Main R code for data analysis

## Supplementary Material

R code containing functions for data analysis

## Supplementary Material

Read me file with details on the R codes

## Supplementary Material

all.B.csv

## Supplementary Material

all.J.csv

## Data accessibility

The data used in this study are available in [8], figures 1 and 2. We also make available a version of these data as an electronic supplementary material.
