# Supplementary material for: An evolutionary model to predict the frequency of antibiotic resistance under seasonal antibiotic use, and an application to Streptococcus pneumoniae
Source: Proc Biol Sci. 2017 May 31;284(1855):20170679. doi: 10.1098/rspb.2017.0679 (PMC5454275; doi:10.1098/rspb.2017.0679)
Supplement: Supplementary Text, Figures and Tables [file rspb20170679supp1.pdf]

An evolutionary model to predict the frequency of antibiotic resistance under seasonal antibiotic use, and an application to *Streptococcus pneumoniae*

Supplementary Information

François Blanquart, Sonja Lehtinen, Christophe Fraser

A. An explicit epidemiological model

Here we show how a specific epidemiological model for the evolution of antibiotic resistance under fluctuating rates of treatment can be linearized to give a simplified dynamics that is a special case of equation (3) in the main text. We assume the focal host population is part of a larger population. Uninfected and infected individuals immigrate into the focal population at a rate m, while individuals from the focal population emigrate at the same rate. We do not model explicitly the dynamics of resistance in other populations. The dynamics of individuals infected with strains 1 and 2 in the focal population reads:

$$\dot{Y_1} = \beta_1 \, X \, Y_1 - \left(u_1 + \alpha_{1,t}\right)Y_1 + m\left(Y_1^{mig} - Y_1\right)$$

$$\dot{Y_2} = \beta_2 \, X \, Y_2 - \left(u_2 + \alpha_{2,t}\right)Y_2 + m\left(Y_2^{mig} - Y_2\right)$$

where the variables are X, the number of uninfected individuals, Y_1, the number of infected with bacterial strain 1, and Y_2, the number of infected with bacterial strain 2. We assume the bacteria population only includes these two strains. For example, strain 1 can be the bacteria resistant to penicillin, and strain 2 the bacteria sensitive to penicillin. For simplicity we will call these strains "resistant" and "sensitive". The parameters are β_1 and β_2, the transmission rates, u_1 and u_2 the natural clearance rates. The rates $\alpha_{1,t}$ and $\alpha_{2,t}$ represent clearance by the various antibiotics prescribed in the population. Specifically, if n antibiotics are prescribed $\alpha_{i,t} = \sum_{j=1}^{n}(1 - f_{i,j,t})\, a_{j,t}$, where $f_{i,j,t}$ is the frequency of resistance to antibiotic j within strain i, potentially evolving as well, and $a_{j,t}$ is the rate of prescription of antibiotic j. The model includes migration at a rate m, and Y_1^{mig} and Y_2^{mig} are the number of individuals of each strain migrating into the focal population. As we are interested in the frequency of resistance among infected individuals, we do not need to specify the exact dynamics of the number of uninfected. The frequency of resistance p evolves as $\dot{p} = \left(\dot{Y_1}Y_2 - Y_1\dot{Y_2}\right)/(Y_1 + Y_2)^2$. This yields:

$$\dot{p} = p\,(1 - p)\big(r_{1,t} - r_{2,t}\big) + M\,(p^{mig} - p)$$

where $r_{i,t} = X\,\beta_i - u_i - \alpha_{i,t}$ for $i \in \{1,2\}$ are the growth rates of the two strains. Generally it is assumed that resistance carries a cost in terms of the transmission or clearance rate, such that depending on antibiotic usage either the sensitive or the resistant type is more fit. The second term represents the impact of migration, proportional to $M = m\,\big(Y_1^{mig} + Y_2^{mig}\big)/(Y_1 + Y_2)$, and tends to bring the frequency of resistance towards the frequency $p^{mig} = Y_R^{mig}/\big(Y_S^{mig} + Y_R^{mig}\big)$.

Assuming no migration (*M = 0*), resistance evolves under temporally fluctuating selection alone. The differential equation can be solved, assuming ecological equilibrium (the number of uninfected is constant), as

$$\text{logit}[p_t] = \text{logit}[p_0] + \big((\beta_1 X - u_1) - (\beta_2 X - u_2)\big)\,t + \int_0^t \big(\alpha_{1,t} - \alpha_{2,t}\big)\,dt$$

The term $\big((\beta_1 X - u_1) - (\beta_2 X - u_2)\big)$ is expected to be negative if strain 1 is resistant and strain 2 is sensitive, and represents the cost of resistance. The term $\int_0^t \big(\alpha_{1,t} - \alpha_{2,t}\big)\,dt$ represents the average impact of fluctuating treatment and is positive. Depending on the balance between these two terms, the resistant or the sensitive strain invades the population, and polymorphism is lost in the long term (1).

With migration, assuming all antibiotics are prescribed at a constant rate equal to their temporal average $\bar{a}_j$, such that clearance by antibiotics is also constant equal to $\bar{\alpha}_i$, p reaches a stable equilibrium value given by:

$$\bar{p} = \frac{-M + \bar{r}_1 - \bar{r}_2 + \sqrt{\big(M - (\bar{r}_1 - \bar{r}_2)\big)^2 + 4\,M\,(\bar{r}_1 - \bar{r}_2)\,p^{mig}}}{2(\bar{r}_1 - \bar{r}_2)}$$

with $\bar{r}_i = X\,\beta_i - u_i - \big(\sum_{j=1}^{n}\big(1 - \bar{f}_{i,j}\big)\,\bar{a}_j\big)$, and the derivative of the differential equation with respect to p, evaluated at $\bar{p}$, is

$$-c = -\sqrt{\big(M - (\bar{r}_1 - \bar{r}_2)\big)^2 + 4\,M\,(\bar{r}_1 - \bar{r}_2)\,p^{mig}} < 0,$$ showing stability of the equilibrium.

We now assume that the fluctuations of antibiotic prescription around the average are small, the fluctuations of resistance around the equilibrium are small, and the fluctuations in the

within-strain levels of resistance around their average are small. Specifically, we rewrite the temporally varying parameters and variables as:

$$p_t = \bar{p}(1 + \delta p_t)$$

$$a_{j,t} = \bar{a}_j\left(1 + \delta \bar{a}_{j,t}\right)$$

$$f_{i,j,t} = \bar{f}_{i,j}\left(1 + \delta f_{i,j,t}\right)$$

where the variables starting with δ represent small temporal fluctuations around the average. The dynamics of the frequency of resistance can be approximated as a first order Taylor series. Actually, only the term of order 1 in the Taylor series needs to be considered, as the term of order 0 cancels by definition of the equilibrium. This yields:

$$\dot{p} \approx -c\,(p_t - \bar{p}) + \sum_{j=1}^{n} \bar{p}\,(1 - \bar{p})\left(\bar{f}_{1,j} - \bar{f}_{2,j}\right)\left(a_{j,t} - \bar{a}_j\right)$$
$$+ \sum_{j=1}^{n} \bar{p}\,(1 - \bar{p})\,\bar{a}_j\left[f_{1,j,t} - f_{2,j,t} - \left(\bar{f}_{1,j} - \bar{f}_{2,j}\right)\right]$$

with

$$c = M - (\bar{r}_1 - \bar{r}_2)(1 - 2\,\bar{p}) = \sqrt{\left(M - (\bar{r}_1 - \bar{r}_2)\right)^2 + 4\,M\,(\bar{r}_1 - \bar{r}_2)\,p^{mig}} > 0$$

The first term is stabilizing selection towards the equilibrium, which depends on the balance between migration *M* (which stabilizes the equilibrium), and directional selection due to differences in the growth rates of the two genotypes (which destabilizes the equilibrium).

The second and third terms represent direct selection for resistance due to antibiotic use. When antibiotic j is prescribed at rates higher than the average ($a_{j,t} > \bar{a}_j$), and strain 1 is more resistant to antibiotic j than strain 2 ($\bar{f}_{1,j} > \bar{f}_{2,j}$), strain 1 increases in frequency at a rate proportional to genetic variance $\bar{p}\,(1 - \bar{p})$ (a classical result in population genetics). Similarly, when resistance to antibiotic j within strain 1 relative to strain 2 is higher than the temporal average ($f_{1,j,t} - f_{2,j,t} > \bar{f}_{1,j} - \bar{f}_{2,j}$), strain 1 increases in frequency at a rate proportional to $\bar{p}\,(1 - \bar{p})\,\bar{a}_j$. The third terms implies that in general, the evolution of a strain is coupled with the evolution of associated resistances within that strain.

For example, if we consider the simple case of two antibiotics, penicillins and macrolides, the evolution of resistance to penicillins and macrolides will be governed by a system of four equations similar to the equations outlined above, describing the changes in frequency in

strains (i) penicillin sensitive / macrolide sensitive, (ii) penicillin sensitive / macrolide resistant, (iii) penicillin resistant / macrolide sensitive, (iv) penicillin resistant / macrolide resistant.

However, if we assume that associations between resistances are relatively constant ($f_{i,j,t} = \bar{f}_{i,j}$), the above equation simplifies to:

$$\dot{p} \approx -c\,(p_t - \bar{p}) + \sum_{j=1}^{n} b_j \left(a_{j,t} - \bar{a}_j\right)$$

with $b_j = \bar{p}\,(1 - \bar{p})\left(\bar{f}_{1,j} - \bar{f}_{2,j}\right)$. This is equation (3) of the paper.

The assumption of constant associations between resistances is motivated by the data analyzed in this paper. We have data on the use of three antibiotics, penicillins (amoxicillin), cephalosporins, and macrolides (azithromycin). This means we should ideally consider the evolution of 2^3=8 strains with the different combinations of sensitivity/resistance, but we only have data on the frequency of resistance to penicillins and macrolides. Associations between resistances may be relatively stable, as for example most of the strains resistant to macrolides are penicillin resistant or intermediate (2) or even multidrug resistant (3).

Simulations:

Supplementary Figure 1 shows simulation of the epidemiological model, together with the approximate linearized system, under seasonal fluctuations in treatment. Coexistence is maintained by immigration from a constant reservoir. The approximation for the dynamics of antibiotic resistance is accurate when antibiotic use fluctuates between 0.5 and 1.5 per month (amplitude = 0.5), and performs relatively well even when antibiotic use fluctuates between 0 and 2 per month (amplitude = 1).

References:

1. Lipsitch M. The rise and fall of antimicrobial resistance. Trends in Microbiology. 2001. p. 438–44.

2. Barkai G, Greenberg D, Givon-Lavi N, Dreifuss E, Vardy D, Dagan R. Community prescribing and resistant Streptococcus pneumoniae. Emerg Infect Dis. 2005;11(6):829–37.

3. Dagan R, Barkai G, Leibovitz E, Dreifuss E, Greenberg D. Will reduction of antibiotic

use reduce antibiotic resistance?: The pneumococcus paradigm. Pediatr Infect Dis J. 2006;25(10):981–6.

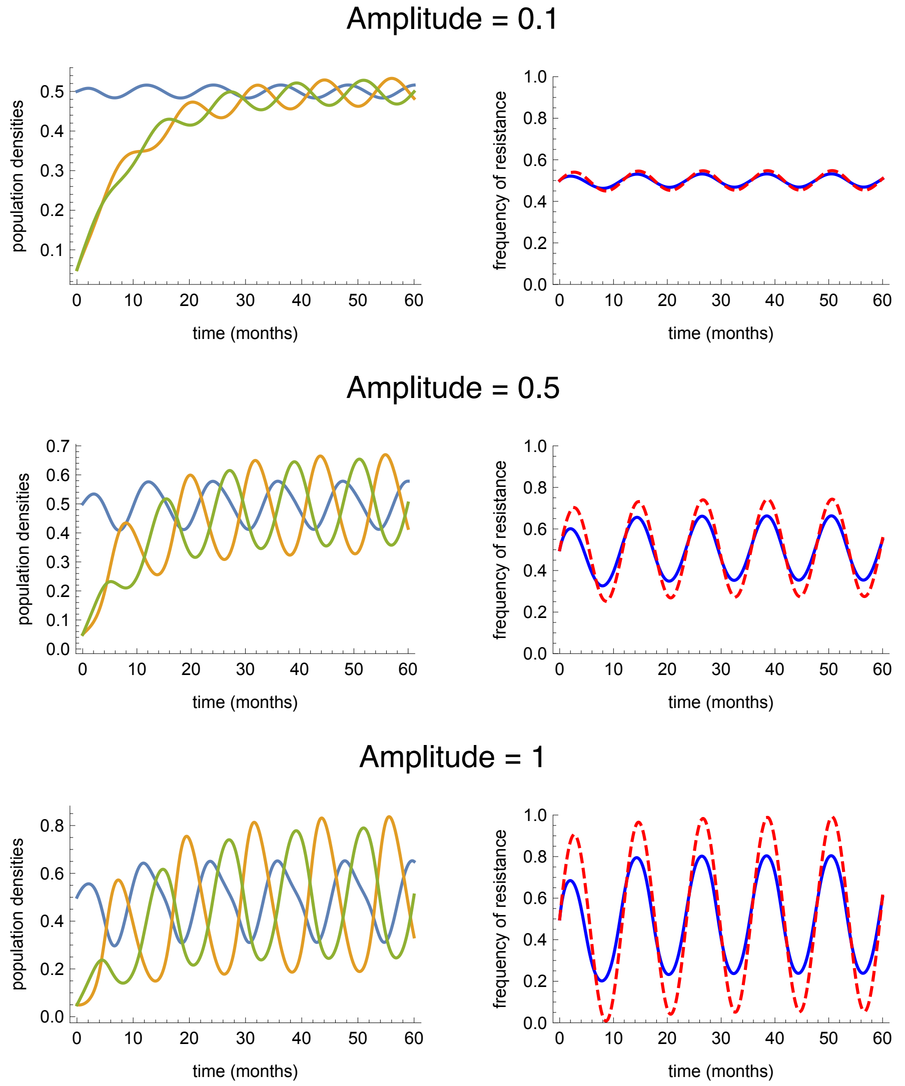

Supplementary Figure 1: Simulations of the epidemiological model, showing the evolution of resistance to an antibiotic resistance under seasonal fluctuations in the prescription of this antibiotic. For the resistant (strain 1), $\alpha_{1,t} = 0$ and for the sensitive (strain 2), $\alpha_{2,t} = \bar{\alpha}(1 + A\cos\left[\frac{2\pi}{12}t\right])$ where t is in months and A is the amplitude, allowed to vary from 0.1 (top panel) to 1 (bottom panel). The left panels show the density of uninfected individuals (blue), of individuals infected by sensitive (orange) and individuals infected by resistant (green). The right panel shows the frequency of resistance (blue) together with the Taylor series approximation (red). Parameters are $\bar{\alpha} = 1\, month^{-1}$, $u = 1\, month^{-1}$, $\beta_1 = 4$, $\beta_2 = 2$, $m = 0.1$, $Y_2^{mig} = 0.5$, $Y_1^{mig} = 0.5$.

B. Expressions for the coefficients in the Fourier transformation:

We describe the temporal change in antibiotic prescription as a sum of sinusoids with different periods, using the Fourier transformation:

$$a_{j,t} - \bar{a}_j = \sum_{k=1}^{T/2} \left(A_{j,k} \cos[\omega_k t - P_{j,k}] \right)$$

The angular frequency of the sinusoids is $\omega_k = 2\pi k/T$ where T is the number of data points, here *T=60* (12 points per year during 5 years). The amplitudes of these sine waves are given by $A_{j,k} = \sqrt{\alpha_{j,k}^2 + \beta_{j,k}^2}$ and the phase differences by $P_{j,k} = \mathrm{atan}\,[\beta_{j,k}/\alpha_{j,k}]$, with:

$\alpha_{j,k} = \frac{2}{T} \sum_{t=1}^{T} a_{j,t} \cos[\omega_k \,(t-1)]$ for $1 \leq k < n$, and $\alpha_{j,n} = \frac{1}{T} \sum_{t=1}^{T} a_{j,t} \cos[\omega_n \,(t-1)]$

$\beta_{j,k} = \frac{2}{T} \sum_{t=1}^{T} a_{j,t} \sin[\omega_k \,(t-1)]$ for $1 \leq k \leq n$

resistance	population	c	b_{amo}	b_{ceph}	b_{azi}	$\bar{p}$	ML	R^2
penicillin	Jewish	180	0.48	-3	0.92	0.35	-146.9	0.43
penicillin	Bedouin	6300	3.9	-31	-1.6	0.23	-159.4	0.18
erythromycin	Jewish	3000	-0.2	-17	17	0.23	-128.5	0.24
erythromycin	Bedouin	18	-0.021	-0.021	0.11	0.15	-163.2	0.28
multidrug	Jewish	2000	0.98	-18	10	0.19	-130.5	0.22
multidrug	Bedouin	13	-0.0048	-0.045	0.083	0.19	-144.1	0.31

Supplementary Table 1. Maximum likelihood parameter estimates for the full model. For each combination of type of resistance and population, we show maximum likelihood estimates of stabilising selection c, direct selection due to amoxicillin, cephalosporin and azithromycin b_{amo}, b_{ceph} and b_{azi}, and the stable frequency of resistance if all antibiotics were used at their average value, $\bar{p}$. Maximum likelihood and the coefficient of determination R^2 of the model are also shown. The intensity of direct selection (the b_i coefficients) has to be interpreted with respect to the strength of stabilising selection c, because $c \gg \omega_j$ for all j, such that the b_i and c are not identifiable.

Period (months)	Amplitude $\boldsymbol{A_{i,j}}$	Phase difference $\boldsymbol{P_{i,j}}$
Jewish, amoxicillin		
Inf	115.2	-
12	39.7	0.05
60	13.6	1.16
Jewish, amoxicillin-clavulanate		
Inf	25.6	-
12	9.8	0.25
60	5.5	1.38
30	3.5	-0.27
5	2.6	0.71
Jewish, cephalosporin		
Inf	13.3	-
60	3.1	2.99
12	2.7	-0.01
30	2.5	-1.72
10	1.9	-2.92
9	1.3	-1.34
Jewish, azithromycin		
Inf	23.1	-
12	11.5	-0.31
60	7.3	-2.41
10	4.9	-2.47
6	4.2	-0.83

Period (months)	Amplitude $A_{i,j}$	Phase difference $P_{i,j}$
Bedouin, amoxicillin		
Inf	184.8	-
12	26.9	0.13
60	22.3	2.12
30	12.3	1.36
9	11.7	0.07
4	11.4	1.6
Bedouin, amoxicillin-clavulanate		
Inf	56.5	-
12	11.4	0.46
30	3.9	0.01
9	3.8	-0.07
60	3.8	2.5
15	3.6	-2.72
Bedouin, cephalosporin		
Inf	38.5	-
60	7.3	2.19
12	6.5	-2.51
10	4	-2.47
Bedouin, azithromycin		
Inf	16.7	-
60	9.9	-3.04
20	3.1	-1.01
12	3	-1.01

Supplementary Table 2. Fourier series decompositions of the fluctuations in antibiotic prescriptions in the Jewish and Bedouin communities, showing the period, amplitude and phase difference of the minimum number of sinusoids of largest amplitude, such that the coefficient of determination is larger than 0.7. Sinusoids are ordered by amplitude, from largest to smallest. The period “Inf” denotes the constant, where amplitude is the temporal average of prescription.

resistance	b_{amo}/c	b_{ceph}/c	b_{azi}/c	$\bar{p}_J$	$\bar{p}_B$	ML	R^2
penicillin	0.0011 [0.00058; 0.0016]	-0.0051 [-0.0076; -0.0027]	0.0019 [-0.00015; 0.004]	0.34	0.24	-323.2	0.35
erythromycin	-0.00069 [-0.0012; -2e-04]	-0.0026 [-0.0048; -0.00034]	0.0071 [0.0055; 0.0084]	0.24	0.16	-294.8	0.33
multidrug	-0.00023 [-0.00068; 0.00028]	-0.0033 [-0.0055; -0.00073]	0.0057 [0.0038; 0.0071]	0.19	0.19	-285.6	0.19

Supplementary Table 3. Maximum likelihood parameter estimates and 95% confidence intervals for the reduced model, when the ratios b_{amo}/c, b_{ceph}/c and b_{azi}/c are estimated on the combined dataset (both Jewish and Bedouin children). For each combination of type of resistance and population, we show maximum likelihood estimates and 95% confidence intervals of the ratios b_{amo}/c, b_{ceph}/c and b_{azi}/c. We also show the maximum likelihood estimate of the stable frequency of resistance $\bar{p}_J$ and $\bar{p}_B$, if all antibiotics were used at their average value, for the Jewish and Bedouin children, the maximum likelihood and the coefficient of determination R^2 of the model.

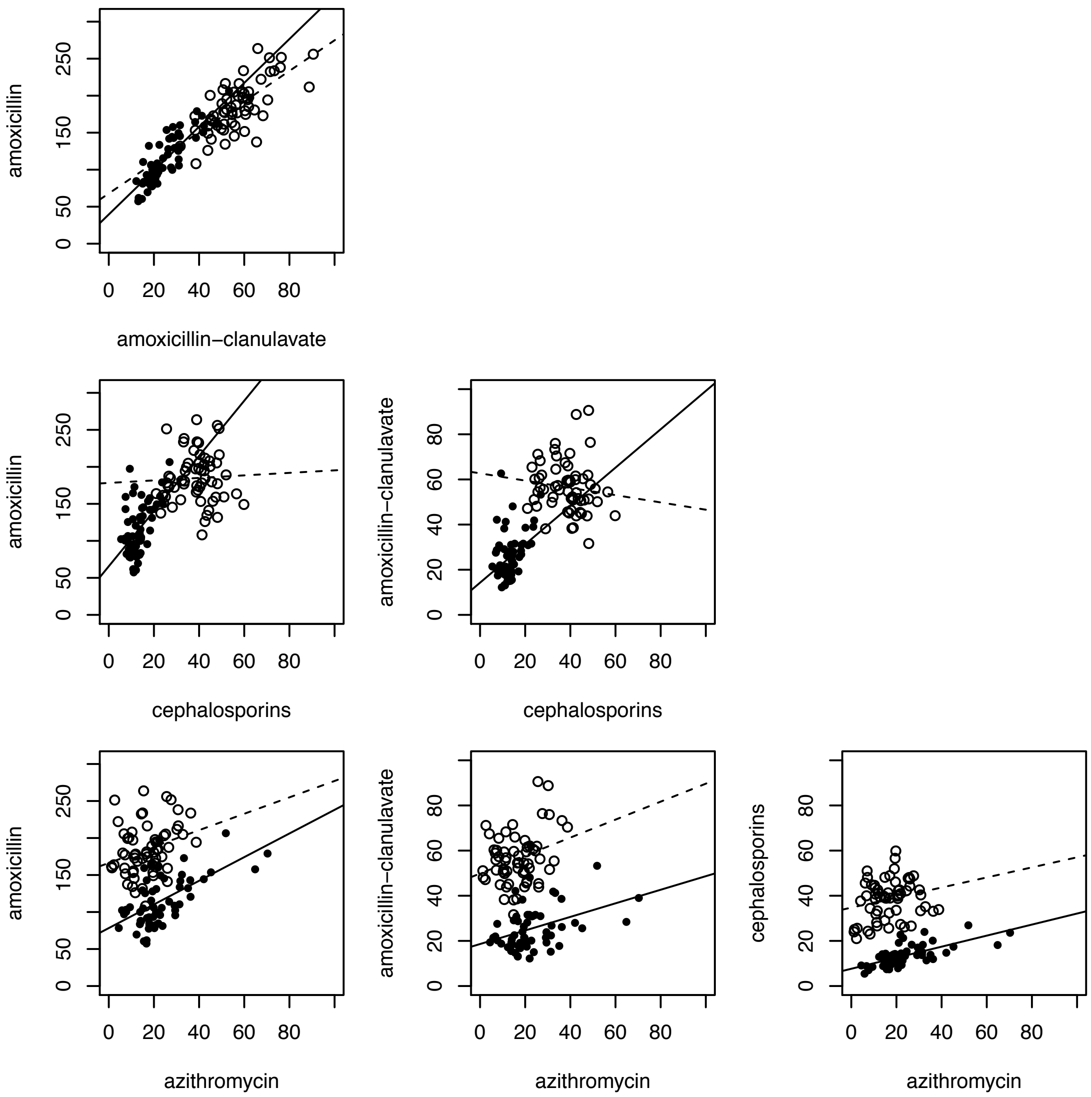

Supplementary figure 2. Temporal correlations in the prescription of different antibiotics, for Jewish (black points) and Bedouin (open points) children, in prescriptions per 1000 children per year. Each point is monthly prescription of a pair of antibiotics. The strongest correlation is between amoxicillin and amoxicillin-clavulanate use. Linear regression lines are shown for the Jewish (plain line) and Bedouin (dashed line) children.

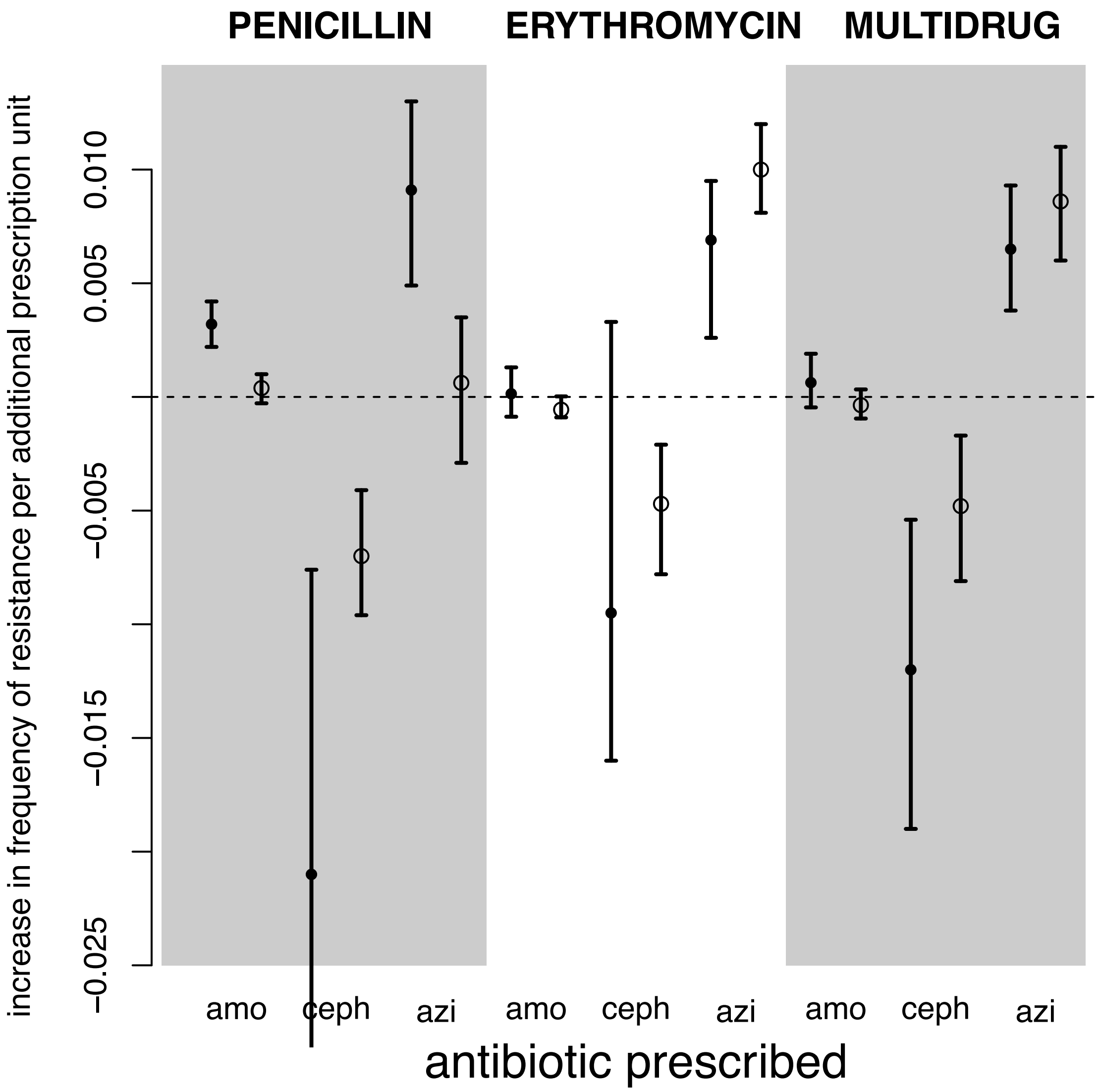

Supplementary Figure 3: Effect sizes and 95% confidence intervals for the ratio b_{amo}/c, b_{ceph}/c and b_{azi}/c, for the population of Jewish children (plain dots) and Bedouin children (open points), when the dynamics of antibiotic prescription are fitted with smooth splines rather than Fourier series. Results are close to those obtained with the Fourier transform (Fig. 3).

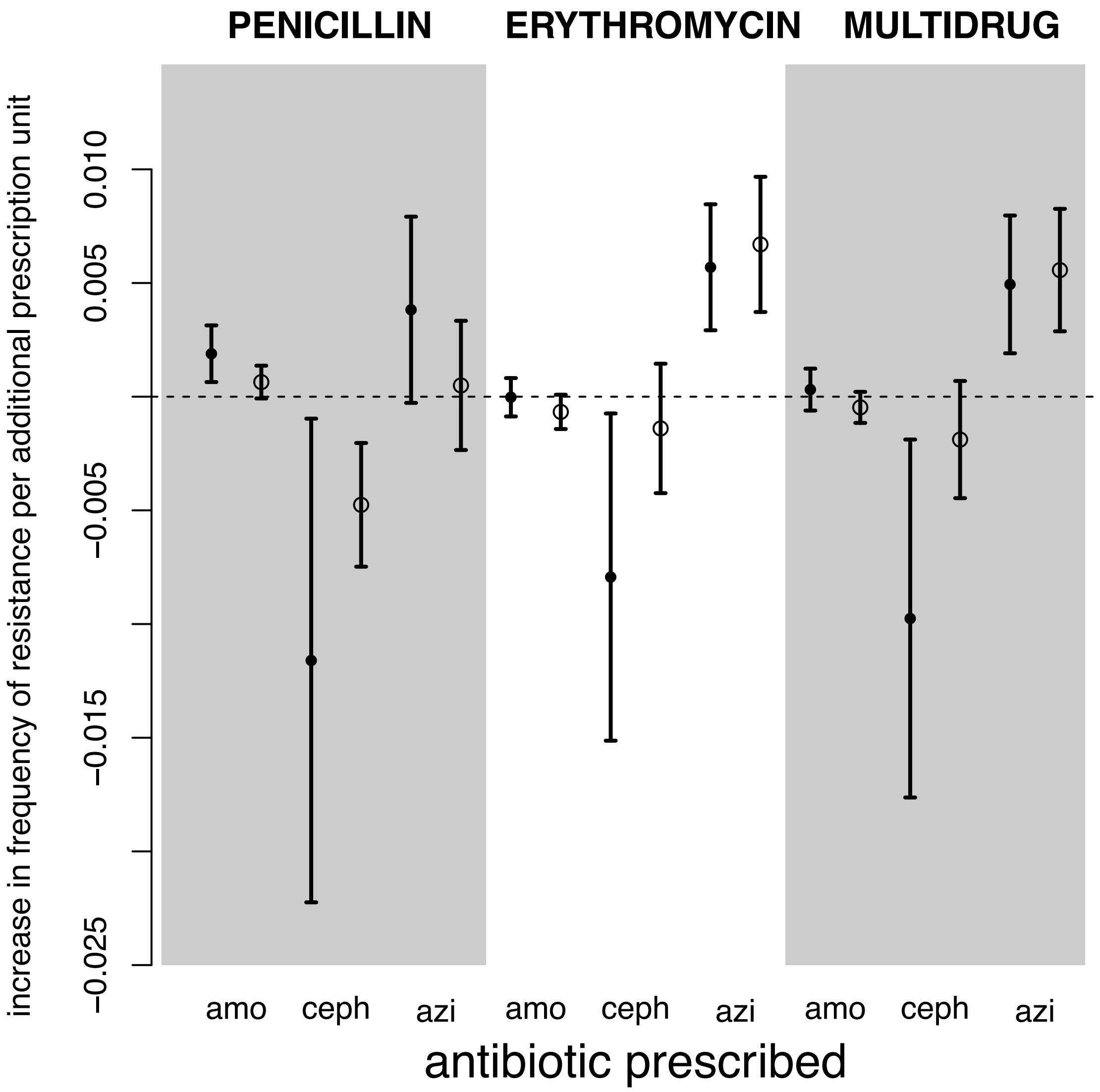

Supplementary Figure 4: Effect sizes and 95% confidence intervals for the ratio b_{amo}/c, b_{ceph}/c and b_{azi}/c, for the population of Jewish children (plain dots) and Bedouin children (open points), estimated by linear modeling. Specifically, for each resistance, we regressed the normalized frequency of resistance onto the normalized antibiotic prescriptions. The model was fitted independently on Jewish and Bedouin children.

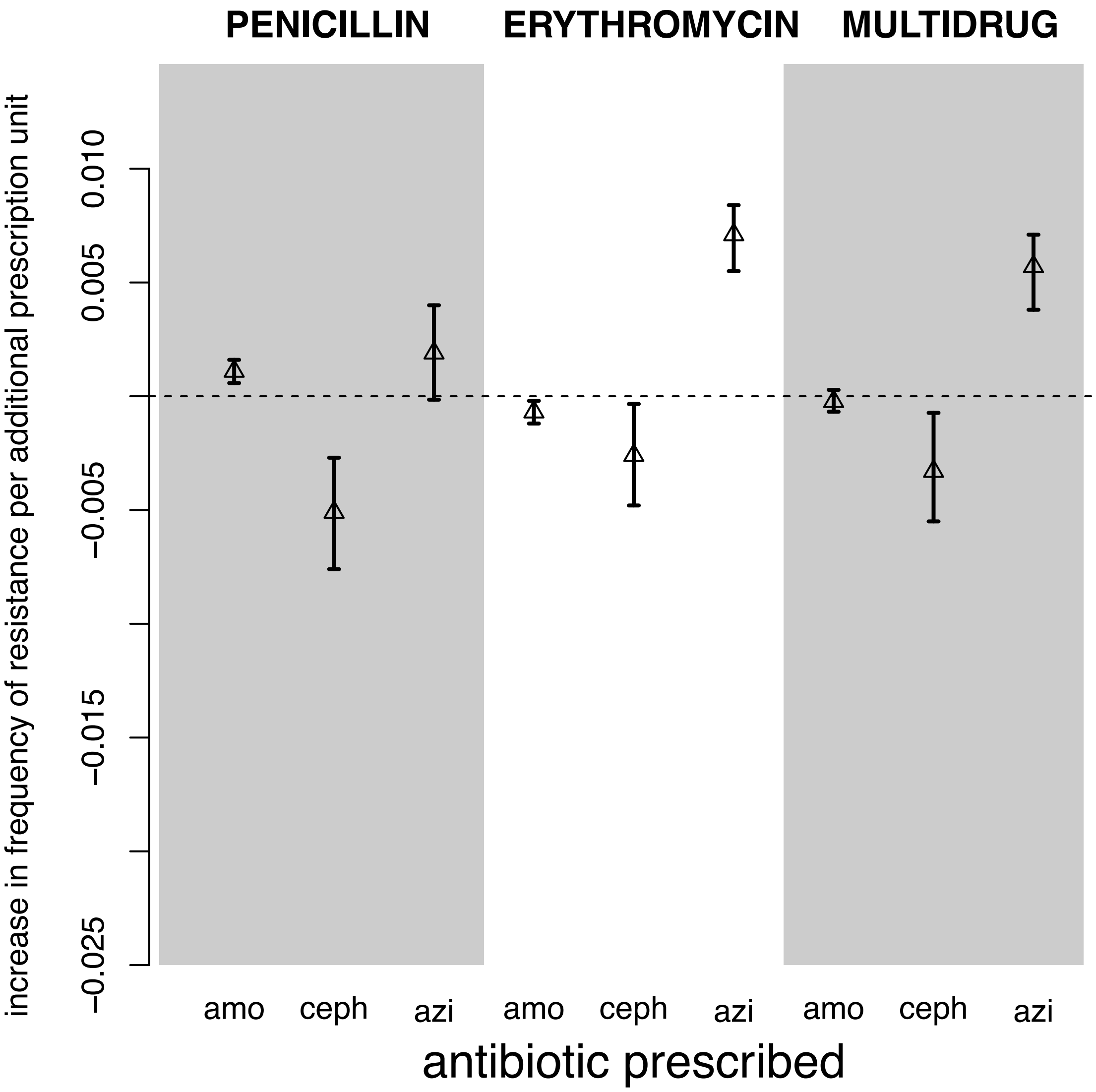

Supplementary Figure 5: Effect sizes and 95% confidence intervals for the ratio b_{amo}/c, b_{ceph}/c and b_{azi}/c, when we infer these coefficients jointly for the Jewish and Bedouin children.
